# A Monte Carlo study comparing dead-time losses of a gamma camera between tungsten functional paper and lead sheet for dosimetry in targeted radionuclide therapy with Lu-177

**DOI:** 10.1007/s12149-024-01987-5

**Published:** 2024-10-01

**Authors:** Kohei Nakanishi, Naotoshi Fujita, Haruna Iwanaga, Yuki Asano, Shinji Abe, Ryuichi Nishii, Katsuhiko Kato

**Affiliations:** 1 Functional Medical Imaging, Biomedical Imaging Sciences, Division of Advanced Information Health Sciences, Department of Integrated Health Sciences, Nagoya University Graduate School of Medicine, 1-1-20 Daiko-Minami, Higashi-Ku, Nagoya, Aichi Japan; 2 Department of Radiological Technology, Nagoya University Hospital, 65 Tsurumai-Cho, Showa-Ku, Nagoya, Aichi Japan; 3 Department of Radiological and Medical Laboratory Sciences, Department of Integrated Health Science, Nagoya University Graduate School of Medicine, 1-1-20 Daiko-Minami, Higashi-Ku, Nagoya, Aichi Japan; 4 Medical Imaging Engineering, Division of AdvancedInformation Health Sciences, Department of Integrated Health Sciences, Biomedical Imaging Sciences, Nagoya University Graduate School of Medicine, 1-1-20 Daiko-Minami, Higashi-Ku, Nagoya, Aichi Japan

**Keywords:** Lu-177, Dead time, Gamma camera, Quantification, Monte Carlo simulation

## Abstract

**Objective:**

Dead-time loss is reported to be non-negligible for some patients with a high tumor burden in Lu-177 radionuclide therapy, even if the administered activity is 7.4 GBq. Hence, we proposed a simple method to shorten the apparent dead time and reduce dead-time loss using a thin lead sheet in previous work. The collimator surface of the gamma camera was covered with a lead sheet in our proposed method. While allowing the detection of 208-keV gamma photons of Lu-177 that penetrate the sheet, photons with energies lower than 208 keV, which cause dead-time loss, were shielded. In this study, we evaluated the usefulness of tungsten functional paper (TFP) for the proposed method using Monte Carlo simulation.

**Methods:**

The count rates in imaging of Lu-177 administered to patients were simulated with the International Commission on Radiological Protection (ICRP) 110 phantom using the GATE Monte Carlo simulation toolkit. The simulated gamma cameras with a 0.5-mm lead sheet, 1.2-mm TFP, or no filter were positioned closely on the anterior and posterior sides of the phantom. The apparent dead times and dead-time losses at 24 h after administration were calculated for an energy window of 208 keV ± 10%. Moreover, the dead-time losses at 24–120 h were analytically assessed using activity excretion data of Lu-177-DOTATATE.

**Results:**

The dead-time loss without a filter was 5% even 120 h after administration in patients with a high tumor burden and slow excretion, while those with a lead sheet and TFP were 0.22 and 0.58 times less than those with no filter, respectively. The count rates with the TFP were 1.3 times higher than those with the lead sheet, and the TFP could maintain primary count rates at 91–94% of those without a filter.

**Conclusions:**

Although the apparent dead time and dead-time loss with the lead sheet were shorter and less than those with TFP, those with TFP were superior to those without a filter. The advantage of TFP over the lead sheet is that the decrease in primary count rates was less.

## Introduction

Targeted radionuclide therapy (TRT) is an effective treatment for various diseases such as metastatic tumors. Several kinds of beta particle emitters have been utilized for therapy in clinical situations. One of them is Lu-177. Treatments with it for pancreatic neuroendocrine tumors (NETs) and castration-resistant prostate cancer have been covered by insurance in recent years in countries including Europe, the USA, and Japan.

Not only does Lu-177 emit beta particles, but it also emits gamma photons. Since the energy of the main gamma photons from Lu-177 is 208 keV, which is suitable for detection with a gamma camera, in-vivo dosimetry is possible. However, there is room for improvement in the quantitative accuracy of the dosimetry; therefore, dosimetry methods with enhanced quantitative performance are being extensively studied worldwide [1]. Dead-time loss is one of the factors affecting dosimetry accuracy. Although the intensity of 208-keV gamma photons from Lu-177 is relatively low at 11% per decay, dead-time loss occurs in dosimetry. This is because gamma photons and X-rays emitted during the decay of Lu-177 at energies lower than 208 keV, such as 113 keV and approximately 55 keV, also contribute to dead-time loss, even when an energy window of 208 keV ± 10% is chosen [2–4]. In addition, the high administered activity contributes to dead-time loss. The dead-time loss is reported to be non-negligible for some patients with a high tumor burden, even if the administered activity is at the typical value of 7.4 GBq [5, 6]. Severe dead-time losses may be a rare occurrence for the administration of 7.4 GBq; however, even 3 days after administration, up to 7.2% of the counts are reportedly lost due to dead time [6]. Moreover, higher activity than 7.4 GBq is administered in personalized treatment; thus, the dead-time loss will be more severe. A study reported that up to 23% and 22% of counts were lost due to dead time at 1 and 3 days after administration in personalized treatment, respectively [6]. It has also been reported that counts were underestimated by more than 10% in approximately 4% of patients at 1 day after administration due to dead-time losses [6], despite the importance of renal dosimetry in improving therapy outcomes and predicting renal toxicity [7]. Moreover, dosimetry based on single time point imaging, such as imaging at 24 h after administration, has been studied, and such dead-time loss could deteriorate the quantification of this type of dosimetry [8–10]. Given the above, the European association of nuclear medicine (EANM) guideline for quantitative measurement with a gamma camera notes that dead-time correction may be necessary, particularly in personalized treatment and measurements taken at early time points after administration in TRT with Lu-177 [4, 11].

The dead time for a specific energy window is referred to as “apparent dead time”. Dead-time loss is corrected using the apparent dead time through the paralyzable model represented by Eq. (1):

1 $${R}_{wo}={R}_{wt} \times {e}^{-{R}_{wt}{\tau }_{w}}$$

where *R*_*wo*_ and *R*_*wt*_ are the observed and true count rates in a certain energy window, respectively, and *τ*_*w*_ is the apparent dead time for a certain energy window. For the correction, the apparent dead time must be measured. Typically, the decaying source method is chosen for this measurement. However, multiple measurements with accurately reproduced experimental setups are necessary; thus, the measurement is burdensome. In addition, a study has reported that the dead times measured under the same conditions varied by more than 20% [12]; thus, accurate dead time measurement is difficult.

In our previous work [13], we proposed a simple method to shorten the apparent dead time and reduce dead-time loss using a thin lead sheet without the need for measuring dead time. However, the thin lead sheet caused a reduction in the primary counts within the set energy window. In this study, we evaluated the usefulness of tungsten functional paper (TFP), which has a lower atomic number than lead, for the proposed method to suppress the reduction in the primary counts using Monte Carlo simulation and compared the results with those obtained using the thin lead sheet.

## Materials and methods

### Method to shorten apparent dead time

The apparent dead time of a gamma camera depends on the window fraction, which is the ratio of count rates in that energy window to the total count rates in the full window, and can be estimated by Eq. (2):

2 $${\tau }_{w}=\frac{\tau }{{wf}^{\eta }}$$

where *τ* is the dead time for full window, *wf* is the window fraction, and *η* is a positive constant, respectively. The value of 1 has been selected for η in Eq. (2) in some studies [3, 5]; however, the optimal value of η was reported to be 1.4 by one study reported [14]. In addition, some studies have indicated that *τ*_*w*_ could not be estimated even when considering both η values of 1 and 1.4 [2, 12]. The studies reported that η values of 1.6 and 1.83 were required, leaving the optimal value of η still a subject of controversy. However, it is clear that the apparent dead time decreases as the window fraction increases.

**Fig. 1:**
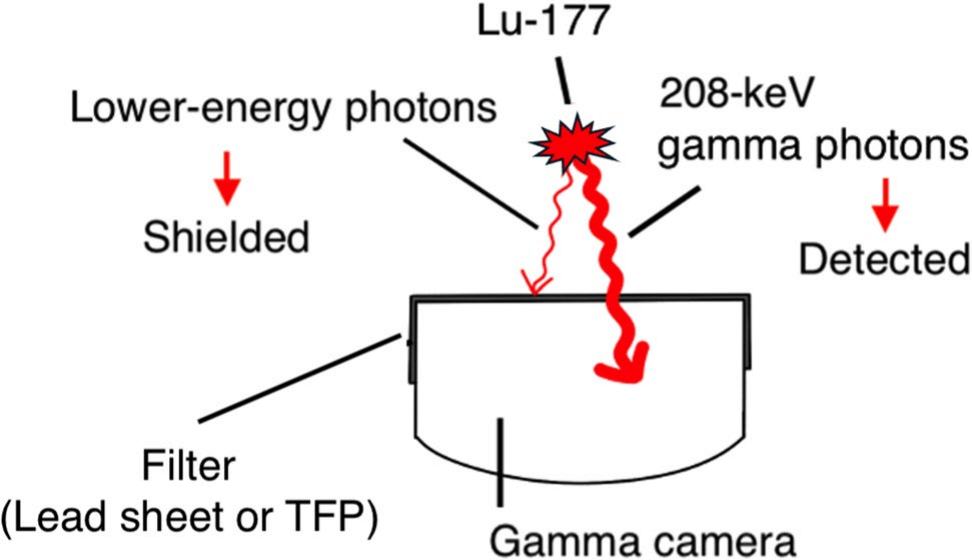
Schematic diagram of our method

We summarized the energies and intensities of the gamma photons and X-rays emitted during the decay of Lu-177 in Table 1. For quantitative measurement of Lu-177, an energy window of 208 keV ± 10% is chosen [2, 4, 5, 15–17]. Lower-energy photons than 208 keV decrease the window fraction. Hence, in our proposed method [13], the lower energy photons are shielded by a thin shielding filter, while detecting 208-keV gamma photons that penetrate the filter as shown in Fig. 1. In this study, a 0.5-mm-thick lead sheet and 1.2-mm TFP were selected as filters for the proposed method because they are commercially available and can be easily acquired. TFP is paper coated with tungsten powder, which accounts for approximately 80% by weight [18]. The elemental compositions of the simulated TFP were hydrogen: 24.2 mol%, carbon: 40.4 mol%, oxygen: 20.2 mol%, and tungsten: 15.2 mol%, which were identical to a previous study [18]. The count rates, apparent dead times, and dead-time losses were compared between using filters and not using a filter with Monte Carlo simulation.

**Table 1:** Energies and intensities of main photons emitted during decay of Lu-177 [19, 20]

	Energy [keV]	Intensity [%/decay]
Gamma photons	208.4	11.0
	113.0	6.4
X-rays	64.9	0.2
	63.2	0.9
	55.8	2.8
	54.6	1.6

### Gamma camera system in Monte Carlo simulation

**Fig. 2:**
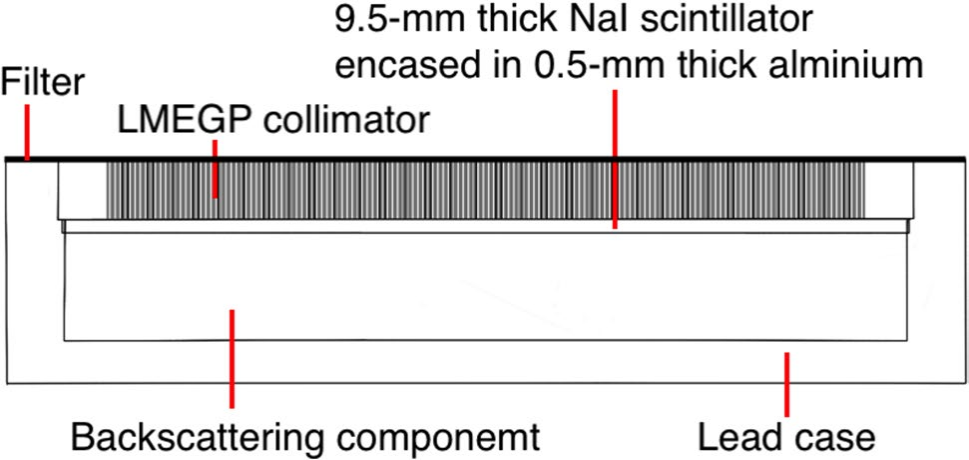
Diagram of simulated gamma camera

We simulated a clinical gamma camera system, the Symbia T6 (Siemens), with a low-medium energy general purpose (LMEGP) collimator using GATE Monte Carlo simulation toolkit. The collimator was attached to a lead case that enclosed a 591 mm × 445 mm × 9.5 mm NaI scintillator in a 0.5-mm-thick aluminum case, with a backscattering component positioned on the back side of the aluminum case, as shown in Fig. 2. The energy resolution of simulated system at 208 keV was 9.4% full width at half maximum (FWHM) [21].

In previous study [13], the simulated observed primary count rate in the 208 keV ± 10% energy window for Lu-177 in a cylindrical water phantom, acquired with and without the shielding filter, was validated well against experimental results obtained in real-world measurements. The differences between simulated and experimental count rates with the 0.5-mm lead sheet was 2.8%, while the differences without filter was 3.6% for count rates below 60 kcps, which includes the expected count rate in patient measurements. This correspondence indicates that the simulation accuracy for dead time and dead-time loss was sufficient. The details of the simulated system and performances were reported in our previous work [13].

### Monte Carlo simulation of dead-time loss in imaging of ICRP voxel phantom at 24 h after administration

**Fig. 3:**
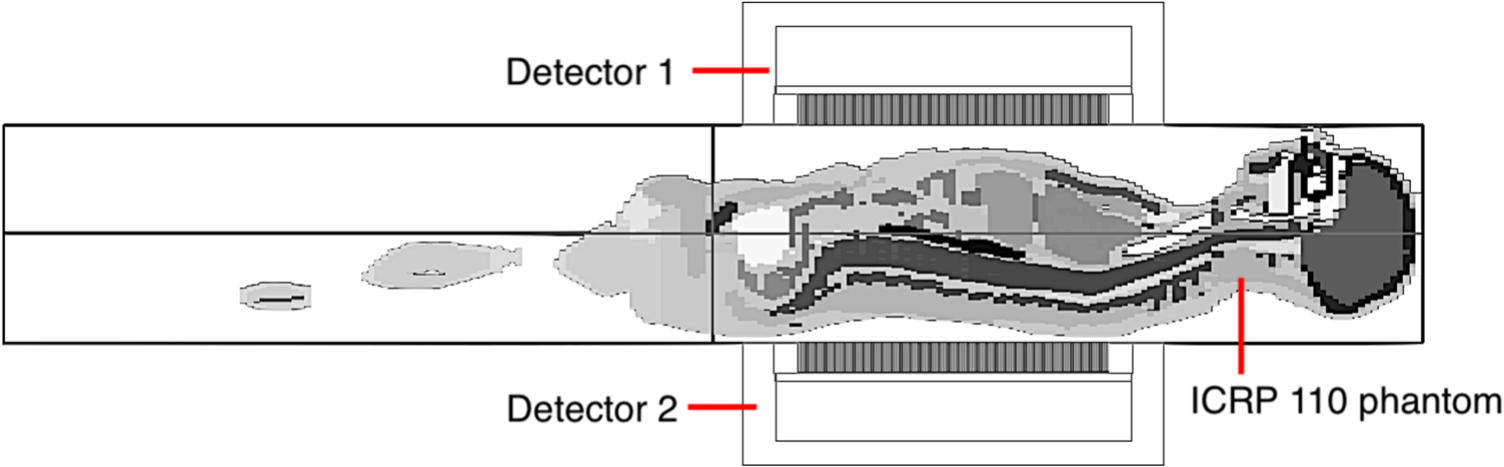
Simulated geometry of Lu-177 imaging in ICRP 110 voxel phantom

The imaging of Lu-177 administered to the patient was simulated using a voxel phantom, which was the male type of International Commission on Radiological Protection (ICRP) 110 phantom [22] as shown in Fig. 3. The activities of Lu-177 were set in the liver, spleen, and kidneys because the uptake of Lu-177-DOTATATE is generally observed in these organs without abnormalities. The values of percent injected activity (%IA) were set identical to those in normal organs 24 h after administration as reported in other studies [23]. However, for the liver, the value for metastatic liver tumors was used since it is a common metastatic organ for NETs. Namely, it is equivalent to having metastatic tumors distributed throughout the entire liver, which is when the dead-time loss would be severe. The simulated injected dose was 7.4 GBq.

The detectors of the simulated gamma camera system were positioned closely on the anterior (detector 1) and posterior sides (detector 2) of the phantom, with the upper abdomen of the phantom in the field of view (FOV). The dead time of the detectors for the full-energy window was set to 0.5 µs based on previously reported values [5, 14, 24]. The true and observed count rates of each detector in the energy window of 208 keV ± 10% were simulated. The simulated measurement time was determined so that the observed total counts would exceed 10 kcounts. The measurement by our proposed method, namely with 0.5-mm lead and 1.2-mm TFP set at the surface of the collimator, was simulated as well as without them.

The primary true and observed count rates in the energy window were calculated using the triple energy window (TEW) method. We set the width of the sub-energy window for the TEW method to 10% [5]. The apparent dead time and dead-time loss in the energy window for each detector was simulated with paralyzable model. We calculated the dead-time loss with 0.5-mm lead and 1.2-mm TFP, as well as without them, using Eq. (3):

3 $$Dead-time \,losses (\text{\%}) =\frac{True \,primary \,count \,rate\,- \,Observed \,primary \,count \,rate}{True \,primary \,count \,rate} \times 100$$

The energy spectra and count rates were recorded every second during the simulated measurements. The window fractions, apparent dead times, and dead-time losses were calculated from each spectrum and count rate. We performed Welch’s *t*-test (α = 0.05) to compare the results with and without filters for each detector, and *p* values were corrected using the Bonferroni method. We summarized the simulated parameters for the measurement described above in Table 2.

**Table 2:** Simulated parameters determined based on other studies

	Energy resolution at 208 keV	Dead time for full-energy window	Energy window	Sub-energy window for TEW
Values	9.4% FWHM	0.5 µs	208 keV ± 10%	10%
References	[21]	[5, 14, 24]	[2–5, 15–17]	[5]

### Analytical assessment combined with Monte Carlo results for dead-time loss at later time points

We analytically assessed the dead-time losses at later time than 24 h after administration. The count rate is proportional to the activity in patients within the FOV of the gamma camera; hence, the true count rate without dead-time losses can be assumed to change proportionally with the retention of activity. The true count rates at later time points were estimated based on the 24-h count rates simulated using Monte Carlo methods and the activity excretion data of Lu-177-DOTATATE reported in another study [25]. The excretion data of patients with slow excretion were used since this study assumed conditions where severe dead-time loss would occur. The observed count rates with and without filter were estimated by the apparent dead times, and the dead-time losses as a function of time points after administration were acquired from the true and observed count rates.

## Results

### Monte Carlo simulation of dead-time loss in imaging of ICRP voxel phantom at 24 h after administration

**Fig. 4:**
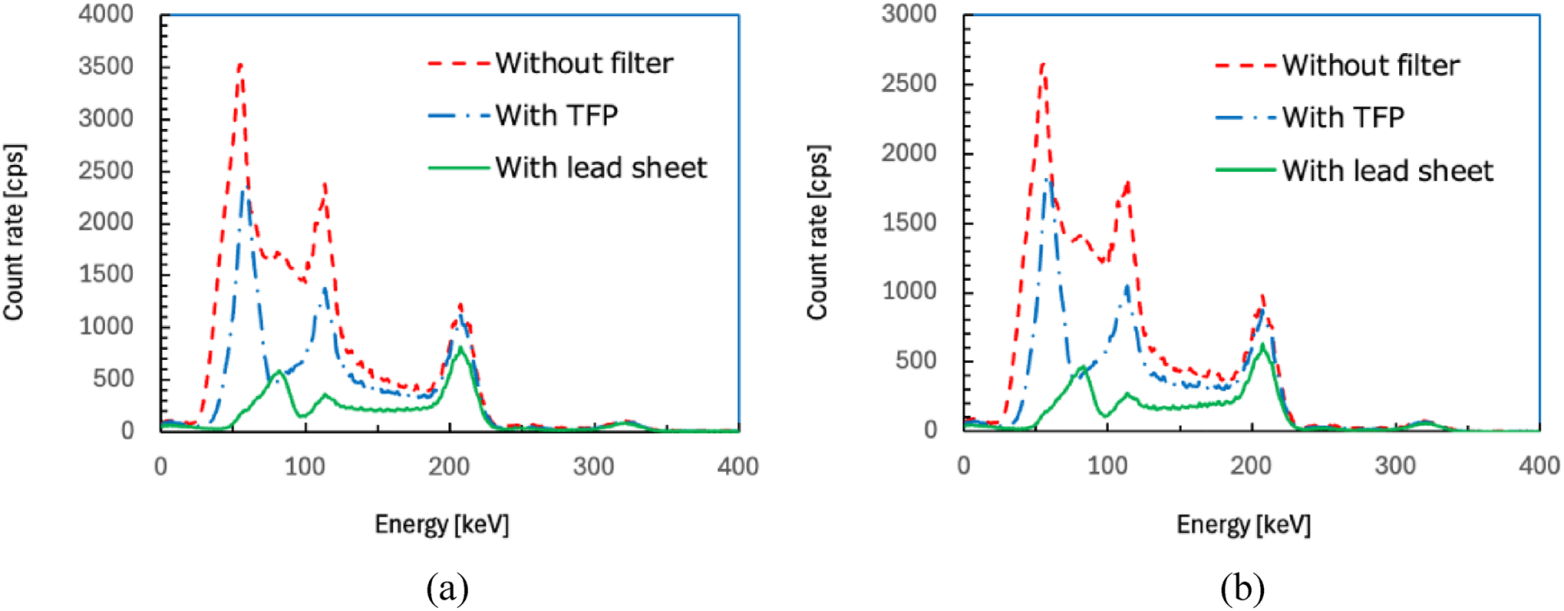
Energy spectra recorded by detectors 1 (**a**) and 2 (**b**) with and without filter

The energy spectra recorded by detectors 1 and 2 are shown in Fig. 4a and b, respectively. The counts of low-energy photons in the energy spectra of both detectors were reduced by the filters.

We summarized the window fractions simulated with and without filter in Table 3. The filters improved the window fraction. The differences in window fractions were statistically significant for both detectors 1 and 2 (*p* < 1.0 × 10^−12^).

**Table 3:** Window fractions with and without filter

	Without filter	With TFP	With lead sheet
Detector 1	0.12 ± 0.00079	0.18 ± 0.00084	0.31 ± 0.0017
Detector 2	0.12 ± 0.00082	0.18 ± 0.0014	0.30 ± 0.0028

The apparent dead times with and without filter are summarized in Table 4. The average dead times between detectors 1 and 2 with lead sheet, TFP, and no filter were 3.0 μs, 5.8 μs, and 9.3 μs, respectively. The dead times were shortened as window fractions increased. The dead times with the lead sheet were one-third shorter than those without the filter, which was consistent with the results acquired using a cylindrical water phantom [13]. We observed statistically significant differences in apparent dead times (*p* < 0.001).

**Table 4:** Apparent dead times with and without filter

	Without filter	With TFP	With lead sheet
Detector 1	8.7 ± 0.43 μs	5.6 ± 0.32 μs	2.9 ± 0.28 μs
Detector 2	9.8 ± 1.4 μs	6.1 ± 0.36 μs	3.2 ± 0.29 μs

**Fig. 5:**
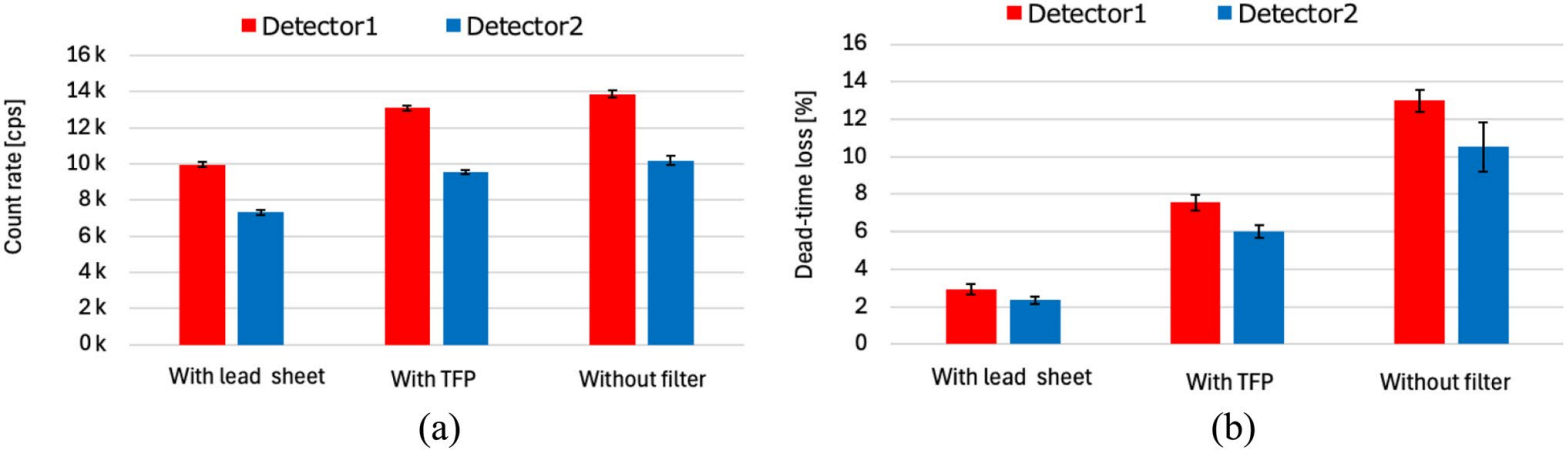
Primary count rates (**a**) and dead-time losses (**b**) with and without filter

Figure 5a shows the primary count rates in the energy window. The error bars represent standard deviations. Statistically significant differences in count rates were observed for both detectors 1 and 2 (*p* < 0.002). The count rates of detector 1 were 36–37% higher than those of detector 2. The count rate of detector 1 with a lead sheet was 2.3% lower than that of detector 2 without a filter; that is, the count rates were at the same level.

Figure 5b shows the dead-time losses with and without filter at 24 h after administration. The filters could statistically significantly reduce dead-time losses (*p* < 0.0002). The dead-time loss of detector 2 without a filter was 3.6 times greater than that of detector 1 with a lead sheet, even though the count rates were at the same level due to the longer apparent dead time of detector 2 without filter.

### Analytical assessment combined with Monte Carlo results for dead-time loss at later time points

**Fig. 6:**
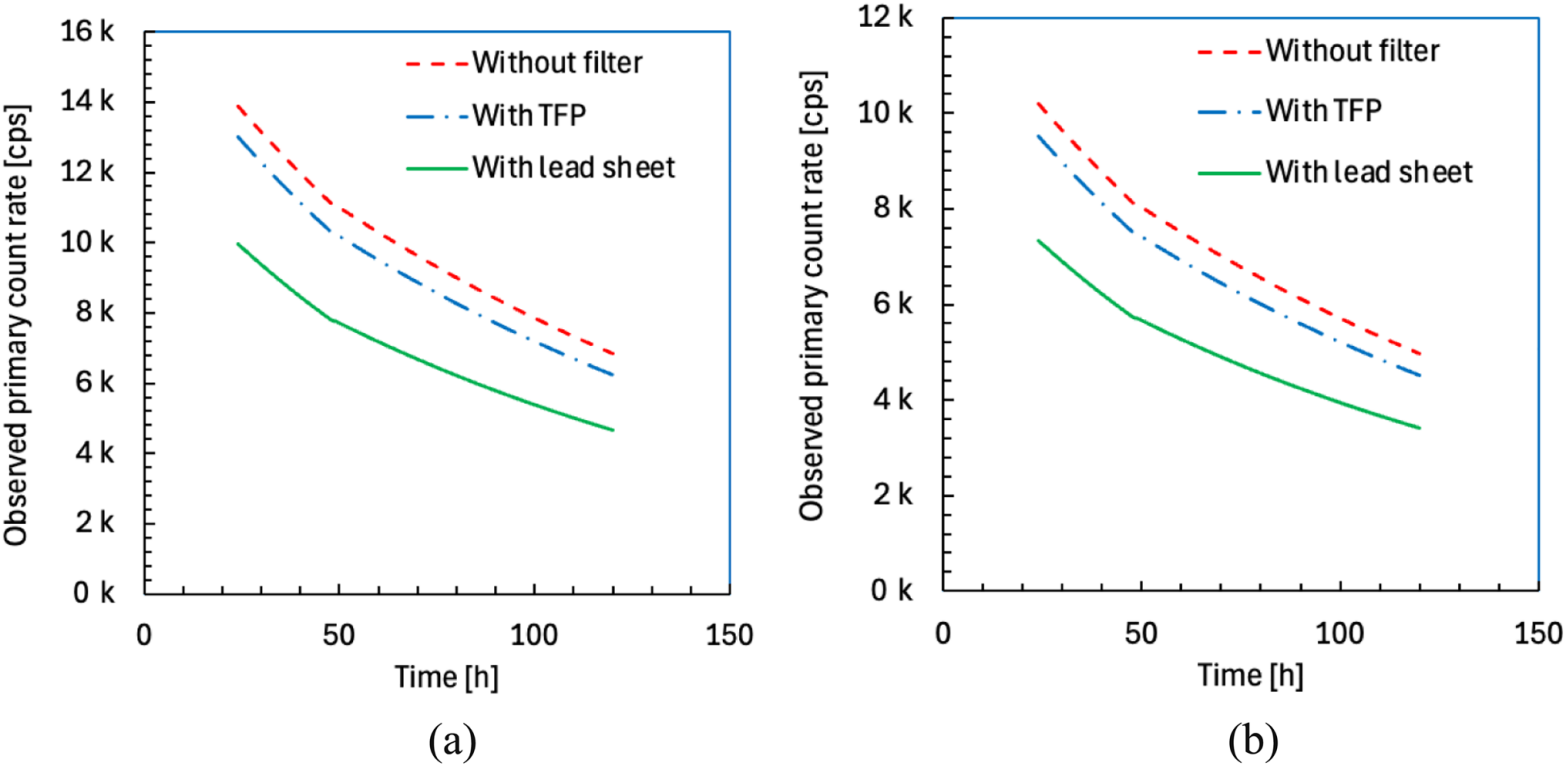
Observed primary count rates of detectors 1 (**a**) and 2 (**b**) as a function of time after administration

The observed primary count rates of detectors 1 and 2 are shown in Fig. 6a and b, respectively. The count rates without filter were 1.4–1.5 times higher than those with a lead sheet and 1.06–1.09 times higher than those with TFP for both detectors.

**Fig. 7:**
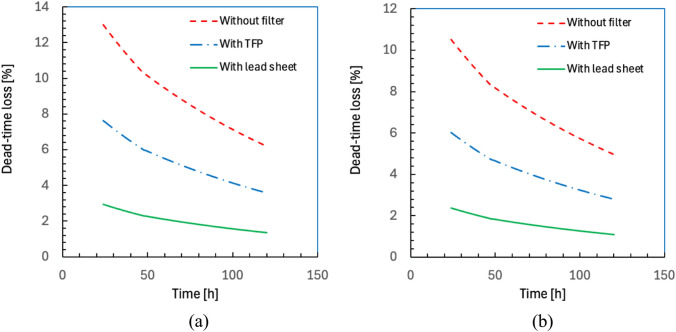
Dead-time losses of detectors 1 (**a**) and 2 (**b**) as a function of time after administration

The dead-time losses of detectors 1 and 2 as a function of time after administration are shown in Fig. 7a and b, respectively. The dead-time loss without a filter was 5% even 120 h after administration in patients with a high tumor burden and slow excretion, while those with a lead sheet and TFP were approximately 0.22 and 0.58 times less than those with no filter, respectively.

## Discussion

We compared the count rates, apparent dead times, and dead-time losses between using filters and not using a filter with Monte Carlo simulation. The apparent dead time with TFP was approximately 0.6 times shorter than without a filter, and the dead-time loss with TFP was 58% of that without a filter. Moreover, the TFP could maintain primary count rates at 91–94% of those without a filter; thus, TFP shows promise for reducing dead-time losses in Lu-177 dosimetry. This may contribute to improved therapy outcomes and more accurate prediction of toxicity due to enhanced renal dosimetry. However, the decrease in counts and the increase in statistical noise in images are drawbacks of our method, as discussed in our previous study [13]. On the other hand, denoising techniques for gamma camera images based on deep learning have been explored [26], and there is potential to address these drawbacks by combining these techniques with our method. This is a goal for our future research toward the clinical implementation of TFP.

The apparent dead time and dead-time loss with the lead sheet were shorter and less than those with TFP. The advantage of TFP is that the decrease in count rates was less compared to lead sheets. The count rates with the TFP were 1.3 times higher than those with the lead sheet.

We simulated a condition where severe dead-time losses would occur, and in such conditions, the lead sheet may be preferred over TFP because the apparent dead time with the lead sheet was shorter. However, such conditions are considered rare for the administration of 7.4 GBq of Lu-177, although they can occasionally arise [6]. In more typical conditions where 1–5% dead-time losses occur, TFP is thought to be more suitable compared with the lead sheet because of the higher primary count rates. Specifically, this condition arises when the true primary count rate without a filter ranges from 1.1 to 5.6 kcounts, with the corresponding observed count rate ranging from 1.09 to 5.3 kcounts.

One key advantage of our method compared to conventional dead-time correction is its simplicity and ease of implementation. Traditional dead-time correction involves complex calculations and requires multiple measurements of count rates with accurate reproduced experimental setup under various radioactivity conditions to determine the dead time [2, 3, 5, 12, 14, 17], none of which are necessary with our approach. Moreover, the uncertainty in calculating dead time affects the accuracy of conventional dead-time correction. In contrast, our method is not affected by this issue, as it does not require the calculation of dead time. Thus, the simplicity and ease of implementation are major advantages of our method, which is why readily available shielding filters, such as lead and TFP, were selected. In addition, our method can reduce pile-up events, which negatively impact spatial and energy resolutions [27]. This proposed method may be useful for enhancing resolution at high count rates.

In this study, the retention of activity in the ICRP phantom at 24 h after administration was 52%IA, which was calculated based on a previous study [23]. On the other hand, in an actual patient with slow excretion, it was reported to be 51%IA in another study [25]. Therefore, the activity in the ICRP phantom was consistent with the condition of an actual patient. Moreover, the simulated dead-time losses without a filter at 24 h and 72 h after administration were 11.7% and 7.8%, respectively, which were the mean values of detectors 1 and 2. Another study reported that the maximum dead-time losses for an administration of 7.4 GBq at days 1 and 3 were 9.8% and 7.2%, respectively [6]. They indicate that our simulated results matched the dead-time losses observed in real-world patients and the accuracy of the simulation was high.

In our simulations, the walls and floor of the examination room were not included, so the simulated conditions did not perfectly match the clinical environment. On the other hand, our simulated results align well with real-world patient results reported in other studies, as described above. Therefore, the impact of differences between the simulated environment and the real clinical setting is likely to be negligible.

As mentioned in Sect. “Gamma camera system in Monte Carlo simulation”, the performance of the simulated gamma camera, both with and without a 0.5-mm-thick lead sheet, was well validated against experimental results. However, the performance with TFP was not validated against experimental results, which is a limitation of this study.

As shown in Fig. 5b, the dead-time losses without a filter were not identical between detectors 1 and 2. Due to differences in dead-time losses between projection angles, the EANM practice guideline recommends dead-time correction for each projection angle, although an average correction for all angles is possible [11]. However, the filters reduced the differences between detectors 1 and 2; thus, our proposed method would be useful for minimizing errors due to differences between projection angles.

The apparent dead times in this study were somewhat longer than those in our previous study. This discrepancy is due to the difference in phantoms used. In the previous study, a cylindrical water phantom was used, whereas in this study, an ICRP voxel phantom was employed. The difference in phantoms led to variations in the window fractions and apparent dead times. Thus, in the real world, the apparent dead time measured using a cylindrical phantom with the decaying source method may not be consistent with the apparent dead time in imaging of actual patient.

Although Symbia T6 gamma camera system was simulated in this study, the apparent dead time increases with a decrease in window fraction for other gamma camera systems as well. Therefore, our method is also useful for other gamma camera systems. However, since each gamma camera system has its own individual dead time [14], the values of apparent dead time and dead-time losses reported in this study may not be applicable to other gamma camera systems, which is a limitation of this study.

In our previous study, we compared 0.5-mm and 1.0-mm-thick lead sheets and selected the 0.5-mm-thick one for this study, as the difference in apparent dead times between the two was small [13]. However, for TFP, we used only a composition identical to that used in other studies [18]; therefore, we did not test TFP with different compositions or thicknesses. The results may be affected if these conditions change. As the thickness of the TFP increases or the effective atomic number rises, the results with TFP will become more similar to those obtained with a lead sheet.

## Conclusion

Although the apparent dead time and dead-time loss with the lead sheet were shorter and less than those with TFP, those with TFP were superior to those without filter. The advantage of TFP over the lead sheet is that the decrease in primary count rates was less.

## Data Availability

The datasets generated during and/or analysed during the current study are available from the corresponding author on reasonable request.

## References

[1] Nautiyal A, Michopoulou S, Guy M. Dosimetry in Lu-177-DOTATATE peptide receptor radionuclide therapy: a systematic review. Clin Transl Imaging. 2024;12(2):157–75. 10.1007/s40336-023-00589-x.

[2] Uribe CF, Esquinas PL, Gonzalez M, Zhao W, Tanguay J, Celler A. Deadtime effects in quantification of ^177^Lu activity for radionuclide therapy. EJNMMI Phys. 2018;5:2. 10.1186/s40658-017-0202-7. 29322344 10.1186/s40658-017-0202-7 PMC5762619

[3] Wicks R, Blau M. The effect of window fraction on the deadtime of anger cameras: concise communication. J Nucl Med. 1977;18:732–5. 874153

[4] Sjögreen Gleisner K, Chouin N, Gabina PM, Cicone F, Gnesin S, Stokke C, et al. EANM dosimetry committee recommendations for dosimetry of 177Lu-labelled somatostatin-receptor-and PSMA-targeting ligands. Eur J Nucl Med Mol Imaging. 2022;49(6):1778–809. 10.1007/s00259-022-05727-7. 35284969 10.1007/s00259-022-05727-7 PMC9015994

[5] Frezza A, Desport C, Uribe C, Zhao W, Celler A, Després P, et al. Comprehensive SPECT/CT system characterization and calibration for ^177^Lu quantitative SPECT (QSPECT) with dead-time correction. EJNMMI Phys. 2020;7:10. 10.1186/s40658-020-0275-6. 32060777 10.1186/s40658-020-0275-6 PMC7021856

[6] Desy A, Bouvet GF, Frezza A, Després P, Beauregard J. Impact of dead time on quantitative ^177^Lu-SPECT (QSPECT) and kidney dosimetry during PRRT. EJNMMI Phys. 2020;7:32. 10.1186/s40658-020-00303-0. 32415492 10.1186/s40658-020-00303-0 PMC7229114

[7] Sundlöv A, Gleisner KS, Tennvall J, Ljungberg M, Warfvinge CF, Holgersson K, et al. Phase II trial demonstrates the efficacy and safety of individualized, dosimetry-based ^177^Lu-DOTATATE treatment of NET patients. Eur J Nucl Med Mol Imaging. 2022;49(11):3830–40. 10.1007/s00259-022-05786-w. 35451612 10.1007/s00259-022-05786-w PMC9399027

[8] Chicheportiche A, Sason M, Zidan M, Godefroy J, Krausz Y, Gross DJ, et al. Impact of single-time-point estimates of ^177^Lu-PRRT absorbed doses on patient management: validation of a trained multiple-linear-regression model in 159 patients and 477 therapy cycles. J Nucl Med. 2023;64(10):1610–6. 10.2967/jnumed.122.264923. 37500259 10.2967/jnumed.122.264923

[9] Willowson KP, Eslick E, Ryu H, Poon A, Bernard EJ, Bailey DL. Feasibility and accuracy of single time point imaging for renal dosimetry following ^177^Lu-DOTATATE (‘Lutate’) therapy. EJNMMI physics. 2018;5:33. 10.1186/s40658-018-0232-9. 30569328 10.1186/s40658-018-0232-9 PMC6300448

[10] Ardenfors O, Nilsson JN, Thor D, Hindorf C. Simplified dosimetry for kidneys and tumors in ^177^Lu-labeled peptide receptor radionuclide therapy. EJNMMI physics. 2022;9(1):44. 10.1186/s40658-022-00473-z. 35723797 10.1186/s40658-022-00473-z PMC9209556

[11] Dickson JC, Armstrong IS, Gabiña PM, Denis-Bacelar AM, Krizsan AK, Gear JM, et al. EANM practice guideline for quantitative SPECT-CT. Eur J Nucl Med Mol Imaging. 2023;50(4):980–95. 10.1007/s00259-022-06028-9. 36469107 10.1007/s00259-022-06028-9 PMC9931838

[12] Heemskerk JWT, Defrise M. Gamma detector dead time correction using Lambert W function. EJNMMI Phys. 2020;7:27. 10.1186/s40658-020-00296-w. 32394021 10.1186/s40658-020-00296-w PMC7214567

[13] Nakanishi K, Fujita N, Abe S, Nishii R, Kato K. A simple method to shorten the apparent dead time in the dosimetry of Lu-177 for targeted radionuclide therapy using a gamma camera. Physica Med. 2024;119: 103298. 10.1016/j.ejmp.2024.103298. 10.1016/j.ejmp.2024.103298 38309102

[14] Silosky M, Johnson V, Beasley C, Kappadath CS. Characterization of the count rate performance of modern gamma cameras. Med Phys. 2013;40: 032502. 10.1118/1.4792297. 23464339 10.1118/1.4792297 PMC3829891

[15] Ardenfors O, Nilsson JN, Thor D, Hindorf C. Simplified dosimetry for kidneys and tumours in ^177^Lu-labeled peptide receptor radionuclide therapy. EJNMMI Phys. 2022;9:44. 10.1186/s40658-022-00473-z. 35723797 10.1186/s40658-022-00473-z PMC9209556

[16] Chicheportiche A, Ben-Haim S, Grozinsky-Glasberg S, Oleinikov K, Meirovitz A, Gross DJ, Godefroy J. Dosimetry after peptide receptor radionuclide therapy: Impact of reduced number of post-treatment studies on absorbed dose calculation and on patient management. EJNMMI Phys. 2020;7:5. 10.1186/s40658-020-0273-8. 31975156 10.1186/s40658-020-0273-8 PMC6977807

[17] Beauregard JM, Hofman MS, Pereira JM, Eu P, Hicks RJ. Quantitative ^177^Lu SPECT (QSPECT) imaging using a commercially available SPECT/CT system. Cancer Imaging. 2011;11:56–66. 10.1102/1470-7330.2011.0012. 21684829 10.1102/1470-7330.2011.0012 PMC3205754

[18] Fujimoto T, Monzen H, Nakata M, Okada T, Yano S, Takakura T, et al. Dosimetric shield evaluation with tungsten sheet in 4, 6, and 9MeV electron beams. Physica Med. 2014;30(7):838–42. 10.1016/j.ejmp.2014.05.009. 10.1016/j.ejmp.2014.05.009 24953537

[19] Deepa S, Sai KV, Gowrishankar R, Rao D, Venkataramaniah K. Precision electron–gamma spectroscopic measurements in the decay of ^177^Lu. Appl Radiat Isot. 2011;69:869–74. 10.1016/j.apradiso.2011.02.012. 21419637 10.1016/j.apradiso.2011.02.012

[20] Sulieman A, Mayhoub FH, Salah H, Al-Mohammed HI, Alkhorayef M, Moftah B, et al. Occupational and ambient radiation exposures from Lu-177 DOTATATE during targeted therapy. Appl Radiat Isot. 2020. 10.1016/j.apradiso.2020.109240. 32819499 10.1016/j.apradiso.2020.109240

[21] Ramonaheng K, van Staden JA, du Raan H. Validation of a Monte Carlo modelled gamma camera for lutetium-177 imaging. Appl Radiat Isot. 2020;163: 109200. 10.1016/j.apradiso.2020.109200. 32561041 10.1016/j.apradiso.2020.109200

[22] Menzel HG, Clement C, DeLuca P. ICRP Publication 110. Realistic reference phantoms: an ICRP/ICRU joint effort. A report of adult reference computational phantoms. Ann ICRP. 2009;39(2):1–164. 10.1016/j.icrp.2009.09.001. 19897132 10.1016/j.icrp.2009.09.001

[23] Brolin G, Gustafsson J, Ljungberg M, Gleisner KS. Pharmacokinetic digital phantoms for accuracy assessment of image-based dosimetry in ^177^Lu-DOTATATE peptide receptor radionuclide therapy. Phys Med Biol. 2015;60:6131–49. 10.1088/0031-9155/60/15/6131. 26215085 10.1088/0031-9155/60/15/6131

[24] Desy A, Bouvet GF, Croteau É, Lafrenière N, Turcotte ÉE, Després P, et al. Quantitative SPECT (QSPECT) at high count rates with contemporary SPECT/CT systems. EJNMMI Phys. 2021;8:73. 10.1186/s40658-021-00421-3. 34718900 10.1186/s40658-021-00421-3 PMC8557232

[25] Levart D, Kalogianni E, Corcoran B, et al. Radiation precautions for inpatient and outpatient 177Lu-DOTATATE peptide receptor radionuclide therapy of neuroendocrine tumours. EJNMMI Phys. 2019. 10.1186/s40658-019-0243-1. 31025215 10.1186/s40658-019-0243-1 PMC6484059

[26] Yang CC, Ko KY, Lin PY. Reducing scan time in ^177^Lu planar scintigraphy using convolutional neural network: a Monte Carlo simulation study. J Appl Clin Med Phys. 2023;24(10): e14056. 10.1002/acm2.14056. 37261890 10.1002/acm2.14056 PMC10562044

[27] Heemskerk JWT, Kreuger R, Goorden MC, et al. Experimental comparison of high-density scintillators for EMCCD-based gamma ray imaging. Phys Med Biol. 2012;57:4545–54. 10.1088/0031-9155/57/14/4545. 22722678 10.1088/0031-9155/57/14/4545

